# Perceived Immune Status and Sleep: A Survey among Dutch Students

**DOI:** 10.1155/2015/721607

**Published:** 2015-09-10

**Authors:** Anouk A. M. T. Donners, Marilou D. P. Tromp, Johan Garssen, Thomas Roth, Joris C. Verster

**Affiliations:** ^1^Division of Pharmacology, Utrecht University, Universiteitsweg 99, 3584 CG Utrecht, Netherlands; ^2^Sleep Disorders and Research Center, Henry Ford Health System, Detroit, MI 48202, USA; ^3^Nutricia Research, Uppsalalaan 12, 3584 CT Utrecht, Netherlands; ^4^Centre for Human Psychopharmacology, Swinburne University of Technology, Melbourne, VIC 3122, Australia

## Abstract

Reduced immune functioning may have a negative impact on sleep and health, and vice versa. A survey among Dutch young adults (18–35 years old) was administered to collect information on perception of reduced immunity and its relationship to sleep disorders, sleep duration, and quality. Sleep disorders were assessed with the SLEEP-50 questionnaire subscales of sleep apnea, insomnia, circadian rhythm disorder, and daily functioning. Dutch young adults (*N* = 574) completed the survey. Among them, subjects (*N* = 209; 36.4%) reported perceived reduced immunity. Relative to those with a normal immune status, subjects reporting reduced immunity had significantly higher scores (*p* = 0.0001) on sleep apnea (2.6 versus 3.6), insomnia (5.1 versus 6.8), and circadian rhythm disorder (2.1 versus 2.7). Subjects reporting reduced immunity also had significantly poorer daily functioning scores (5.4 versus 7.6, *p* = 0.0001). No differences were observed in total sleep time, but those reporting reduced immunity had significantly poorer ratings of sleep quality (6.8 versus 7.2, *p* = 0.0001). Our findings suggest that perceived reduced immunity is associated with sleep disturbances, impaired daily functioning, and a poorer sleep quality. Experimental studies including the assessment of immune biomarkers and objective measures of sleep (polysomnography) should confirm the current observations.

## 1. Introduction

A bidirectional relationship has been suggested between sleep and immune functioning [1]. Sleep and a well-functioning balanced immune system are both essential to maintain a normal health status. Research has shown that reduced total sleep time or sleep quality is related to reduced immune functioning and thus increases susceptibility to disease [2, 3]. Conversely, sleep quality and total sleep time are impaired during infection [4]. Sleep loss has a significant impact on quality of life and poor sleep quality is related to a variety of diseases [5], has a significant impact on total health care costs, and is a major cause of work absenteeism [6]. Correspondingly, sufficient sleep time is associated with better immune functioning and positive health outcomes [3, 7, 8].

Results from several studies, including objective measurements such as actigraphy or determining biomarkers of immune functioning, support the existence of the bidirectional relationship between sleep and immune functioning. For example, Orzech et al. demonstrated the relationship between sleep, immune functioning, and susceptibility to disease [9]. They evaluated sleep in (*N* = 56) adolescents via actigraphy and assessed health status with weekly interviews. The authors found that a shorter total sleep time was associated with more frequently experiencing acute illnesses such as common cold, flu, gastroenteritis, and other common infectious diseases that are a result of reduced immune functioning. Similarly, Cohen et al. described that lower sleep efficiency and shorter sleep duration in the weeks before exposure to a rhinovirus are associated with lower immune resistance and an almost three times increased risk to develop a common cold [2]. In line, Drake et al. showed that having a common cold has detrimental effects on sleep, psychomotor performance, and daytime alertness [10]. Although common cold seems a relative harmless medical condition, it is the most common complaint in general practice [11, 12], with an underestimated economic impact in terms of absenteeism and reduced work productivity [6].

Several studies have investigated the relationship between sleep loss and immune functioning [7, 13–17]. These studies revealed that sleep loss and poor sleep quality are associated with dysregulation of immune functioning. For example, sleep loss has been related to increases of proinflammatory cytokines and reduced natural killer cell activity [18]. Proinflammatory cytokines interleukin- (IL-) 1, IL-6, and tumor necrosis factor-*α* (TNF-*α*) are involved in regulating both sleep and immune function [19–22]. For example, in healthy men, Dimitrov et al. [22] demonstrated a significant decrease in TNF-*α* concentrations during sleep when compared to being awake at night, and Ruiz et al. [23] found an increase in leukocytes, neutrophils, and cluster of differentiation (CD4+) concentrations during 2 nights of total sleep deprivation. However, other studies did not report changes in IL-1*β*, IL-6, and TNF-*α* in relation to sleep and sleep deprivation [24–26].

Whereas the majority of studies focused on the relationship between immune functioning and sleep quality and total sleep time [21], several other studies examined immune functioning in patients with sleep apnea. A recent meta-analysis of 51 studies identified several inflammatory markers that are common in patients with sleep apnea, including elevated serum concentrations of C-Reactive Protein, TNF-*α*, IL-6, IL-8, intercellular adhesion molecule (ICAM), vascular cell adhesion molecule (VCAM), and selectins [27]. Effective treatment of sleep apnea with continuous positive airway pressure has been shown to decrease the elevated IL-6 serum concentrations [28]. The presence and severity of other sleep disorders remain understudied in relation to immunocompetence. In patients with insomnia, elevated levels of C-Reactive Protein were found, which are also present during inflammation [29, 30]. Irwin et al. described increased levels of C-Reactive Protein in patients suffering from insomnia, which reduced to normal levels after effective treatment [31], whereas Savard et al. observed reduced numbers of lymphocyte subpopulations (i.e., CD3+, CD4+, and CD8+ cells) in chronic insomniacs [32]. These examples all illustrate objective immune alterations in people suffering from insomnia.

It can be questioned however whether patients are aware of these objective immune alterations and to what extent they have an impact on related health behavior. Although it is extremely important to have objective assessments of immune functioning, people are often judging their health status based on their* perceived* immune status (i.e., feelings of reduced resistance). Similarly, although sleep may be objectively disturbed, people only visit their physician if it is experienced as such. It is based on this subjective health status that people judge whether or not to visit their physician and decide if they are capable of going to work or should call in sick. Hence, it can be argued that the subjective health experience is a critical determinant of people's choices regarding absenteeism and making use of the healthcare system. Up to now, data relating perceived immune status to sleep quality and duration is scarce. Therefore, the aim of the current study is to explore the relationship between perceived immunocompetence, sleep disorders, and daily functioning. More specifically, it is investigated whether perceived reduced immune functioning reduces sleep quality and total sleep time, results in higher severity scores on sleep disorder scales for insomnia, apnea, and circadian rhythm disorder (CRD), and has a negative impact on daily functioning.

## 2. Methods

A survey among Dutch young adults (18–35 years old) on immunity, sleep disorders, daily functioning, sleep duration, and quality was administered. Most of the participants were recruited at the campus of Utrecht University, Netherlands. Informed consent was obtained before starting the survey either in person or online through http://thesistools.com/. Demographic data including age, gender, height, and weight were collected. Participants indicated whether they were experiencing reduced immunity or perceived a normal health status in the past four weeks. To examine the presence and severity of subjective sleep complaints over the past four weeks, four of the nine subscales (26 items) of the Dutch SLEEP-50 questionnaire were completed [33]. The four subscales include sleep apnea (eight items), insomnia (nine items), CRD (three items), and impact of sleep complaints on daytime functioning (six items). Because sleep disorders can have a profound impact on daily functioning, the corresponding subscale was included as well. Each question was scored on a 4-point scale: 1 (not at all), 2 (somewhat), 3 (rather much), and 4 (very much). Cut-off values for a positive screen for sleep disorders include ≥15 on sleep apnea items (sensitivity = 85%, specificity = 88%), ≥19 on insomnia items (sensitivity = 71%, specificity = 75%), ≥8 on CRD items (sensitivity = 83%, specificity = 69%), and ≥15 on daily functioning (sensitivity = 84%, specificity = 77%) [33]. In addition to the SLEEP-50 questionnaire subscales, prior night's total sleep time was recorded, and an overall sleep quality score ranging from 0 (very poor) to 10 (very good) was obtained.

Statistical analyses were conducted using the Statistical Package for the Social Sciences (SPSS) version 20 (IBM SPSS Statistics). Mean and standard deviation (SD) were computed for each variable and it was determined if scores were normally distributed. SLEEP-50 subscale scores, sleep duration, and quality of subjects who reported reduced immunity were compared to those who reported a normal health status. As the SLEEP-50 data were not normally distributed, the Independent Samples Mann-Whitney *U* Test was used for the statistical analyses. Results were considered significant if *p* < 0.05.

## 3. Results

*N* = 574 Subjects (*N* = 574) completed the survey. Their mean age was 22.3 years old and 68.5% of the sample was women. Perceived reduced immunity was reported by subjects (*N* = 209; 36.4%). Subjects with perceived reduced immunity were significantly older (22.5 versus 21.9 years old, *p* = 0.024), smoked significantly more cigarettes per day (1.8 versus 0.7 cigarettes, *p* = 0.001), and consumed significantly more alcohol per week (10.5 versus 8.1 drinks, *p* = 0.009) when compared to subjects who reported a normal health status. Subjects (*N* = 13; 2.2%) were screened positive for having a sleep disorder.

**Figure 1:**
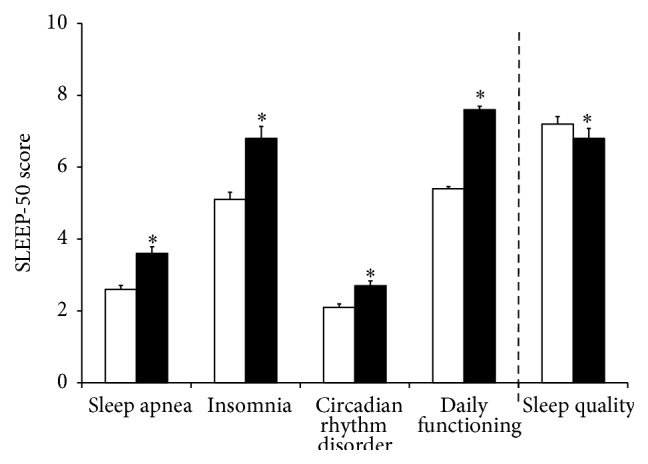
Mean (SE) scores of subjects with perceived normal health status (white bars) or perceived reduced immune functioning (black bars) on sleep disorders, sleep quality, and daily functioning. Significant differences (*p* < 0.05) are indicated by an asterisk.

Significant differences in SLEEP-50 scale scores were found between subjects who reported a normal health status and those reporting reduced immunity (see Figure 1).

Subjects reporting reduced immunity scored significantly higher (*p* = 0.0001) on the subscales of sleep apnea (2.6 versus 3.6), insomnia (5.1 versus 6.8), and circadian rhythm disorder (2.1 versus 2.7). Subjects reporting reduced immunity also reported significantly poorer daily functioning related to their sleep difficulties (5.4 versus 7.6, *p* = 0.0001). No significant differences were observed in total sleep time (*p* = 0.733), but those reporting reduced immunity had a significantly poorer rating of sleep quality (6.8 versus 7.2, *p* = 0.0001).

## 4. Discussion

This study confirmed an association between sleep and perceived immune functioning. Although no effects on total sleep time were observed, in line with previous studies subjects with a perceived reduced immunocompetence reported poorer sleep quality. In addition, these subjects scored significantly higher on SLEEP-50 subscales screening for sleep apnea, insomnia, and circadian rhythm disorders. These findings were reflected in significantly poorer scores on daily functioning in those reporting reduced immune functioning.

The importance of the relationship between sleep and immune functioning should not be underestimated. Potentially, if patients with insomnia are effectively treated this would reduce their susceptibility for immune-related diseases and improve daytime functioning. Conversely, immune status is likely to improve by improving overall sleep. In this context, research has shown that increasing sleep time reduces the susceptibility to disease per se [34]. Enforced by the demands of our 24 h society, the average duration of a normal night of sleep (seven to eight hours) has been reduced by up to two hours per day over the past 35 years [35, 36]. Over the same time period, a significant increase in prevalence rates of immune-related diseases has been reported [37], which makes it tempting to link both developments to each other. However, many other factors are postulated for the increase in incidence and severity of immune-related disorders such as dietary changes, life style changes, hygiene hypotheses, and microbiome diversity. The corresponding impact on quality of life and health care demands is evident [5].

Several limitations of this pilot study need to be addressed. First, the study was conducted only among young adults. As this is a relative healthy segment of the population, SLEEP-50 scores of the vast majority of these participants did not reach the cut-off scores to be screened positive for sleep apnea, insomnia, and circadian rhythm disorder. It can be questioned if this sample of convenience is representative for the general population. Generally individuals in this group sleep less and have a lower prevalence of sleep disorders. Results may be different for groups of different age and socioeconomic status. Second, it should be realized that the SLEEP-50 is a screening instrument and is not meant to formally diagnose sleep disorders. Including polysomnography in these studies would further extend our knowledge. Future research should therefore replicate our findings including a formal diagnosis of sleep disorders. Third, immune status was assessed by a single item question that could be answered by yes or no. It would have been more informative to use questions providing more details on the nature of perceived immune status. Then one could differentiate between subjects with various severity levels of reduced immunocompetence and further explore their relationship with sleep by correlating these severity scores with scores on the SLEEP-50 subscales. Finally, no immune biomarkers were assessed in this study to objectively confirm the reported perceived immune status. While all of these limitations are important they all would result in decreased sensitivity to changes in both sleep and immune function. Despite this lack of sensitivity a significant relationship was found between sleep and immune function.

## 5. Conclusions

This study showed that subjects who perceive reduced immune functioning have significant higher scores on scales assessing sleep apnea, insomnia, and CRD. They also report significantly poorer sleep quality and have significant lower scores on daytime functioning. Future studies with formal diagnosis of sleep disorders and assessment of immune biomarkers should confirm these findings.
